# Genome sequence and description of *Pantoea septica* strain FF5

**DOI:** 10.1186/s40793-015-0083-0

**Published:** 2015-11-14

**Authors:** Cheikh Ibrahima Lo, Roshan Padhmanabhan, Oleg Mediannikov, Thi Tien Nguyen, Didier Raoult, Pierre-Edouard Fournier, Florence Fenollar

**Affiliations:** Aix-Marseille Université, URMITE, UM63, CNRS 7278, IRD 198, Inserm U1095, Faculté de médecine, 27 Boulevard Jean Moulin, 13385 Marseille cedex 05, France; Campus international UCAD-IRD, Dakar, Senegal; Special Infectious Agents Unit, King Fahd Medical Research Center, King Abdulaziz University, Jeddah, Saudi Arabia

**Keywords:** *Pantoea septica*, Genome, Taxonogenomics, Culturomics, Senegal

## Abstract

Strain FF5 was isolated from the skin flora of a healthy Senegalese 35-year-old woman. This strain was identified as belonging to the species *Pantoea septica* based on *rpoB* sequence identity of 99.7 % with *Pantoea septica* strain LMG 5345^T^ and a highest MALDI-TOF-MS score of 2.3 with *Pantoea septica.* Like *P. septica*, this FF5 strain is a Gram-negative, aerobic, motile, and rod-shaped bacterium. Currently, 17 genomes have been sequenced within the genus *Pantoea* but none for *Pantoea septica*. Herein, we compared the genomic properties of strain FF5 to those of other species within the genus *Pantoea*. The genome of this strain is 4,548,444 bp in length (1 chromosome, no plasmid) with a G + C content of 59.1 % containing 4125 protein-coding and 68 RNA genes (including 2 rRNA operons). We also performed an extensive phenotypic analysis showing new phenotypic characteristics such as the production of alkaline phosphatase, acid phosphatase and naphthol-AS-BI-phosphohydrolase.

**Electronic supplementary material:**

The online version of this article (doi:10.1186/s40793-015-0083-0) contains supplementary material, which is available to authorized users.

## Introduction

*Pantoea septica* Brady et al. 2010 was first isolated from a human stool sample in New Jersey USA [1]. *Pantoea septica* strain FF5 (= CSUR P3024 = DSM 27843) was cultivated from the skin of a healthy Senegalese woman [2]. To date, the genus *Pantoea* consists of 22 species and 2 subspecies [3, 4] and no genome had been described for *Pantoea septica* when this paper was written. *Pantoea* species have been isolated mostly from the environment, particularly from plants, seeds and vegetables, several being phytopathogenic [5]. Some species such as *P. agglomerans**,**P. septica* and *P. eucrina* are also frequently isolated from humans in whom they can cause opportunistic infections [1–6].

We provide here a summary classification and a set of features for *Pantoea septica* strain FF5, together with the description of the complete genomic sequence and annotation.

## Organism information

### Classification and features

**Fig. 1:**
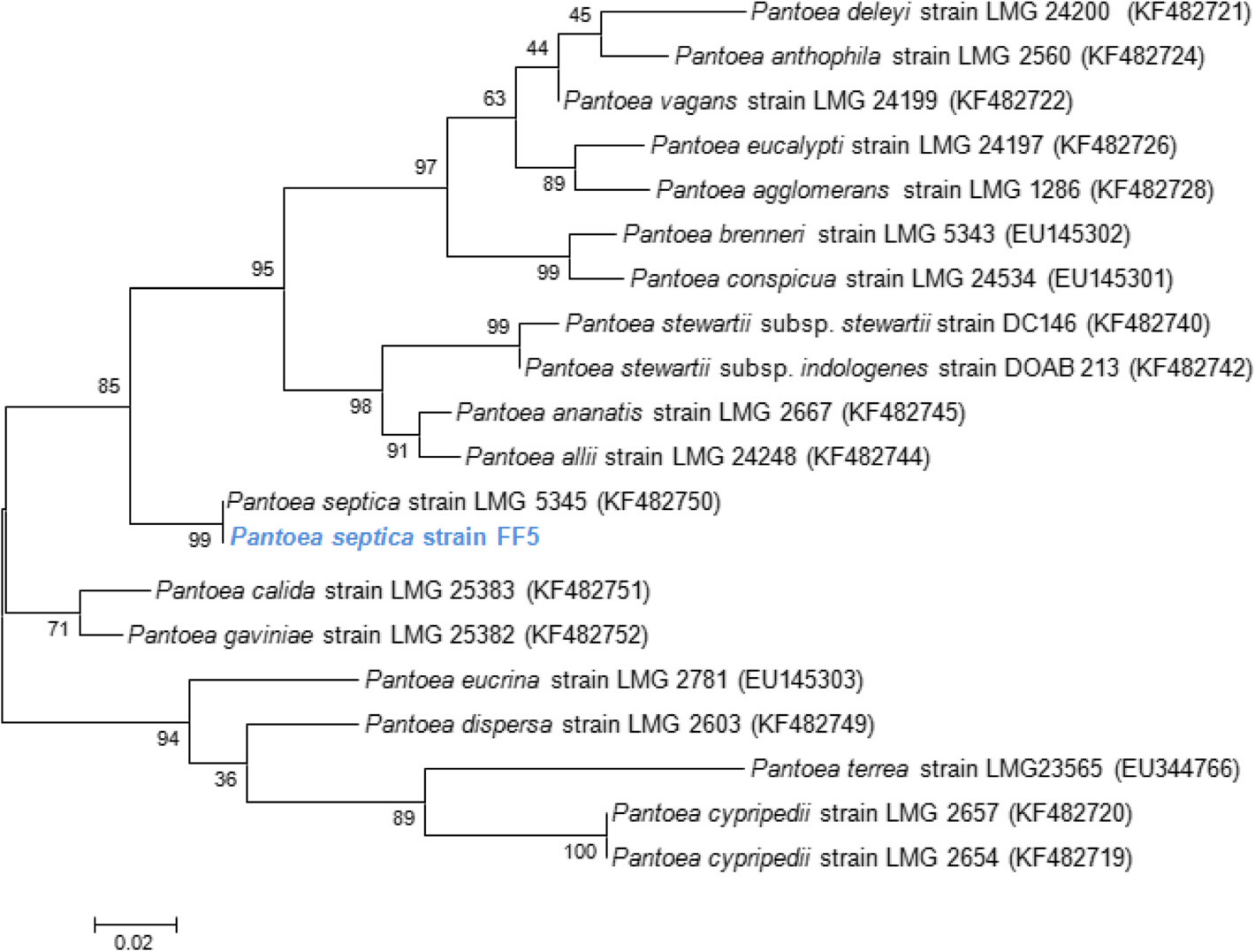
Phylogenetic tree showing the position of *Pantoea septica* strain FF5 relative to other strains within the genus *Pantoea*. The *rpoB* sequences were aligned using MUSCLE [31], and the phylogenetic tree was inferred using the Maximum Likelihood method with Kimura 2-parameter model from MEGA software. Numbers at the nodes are percentages of bootstrap values obtained by repeating the analysis 1,000 times to generate a majority consensus tree. The scale bar represents a rate of substitution per site of 0.02

A skin sample was collected with a swab from a healthy Senegalese volunteer living in Dielmo (a rural village in the Guinean-Sudanian area in Senegal) in December 2012 (Table 1). This 35-year-old woman was included in a research project that was approved by the Ministry of Health of Senegal, the assembled village population and the National Ethics Committee of Senegal (CNERS, agreement numbers 09–022), as published elsewhere [7]. Strain FF5 (Table 1) was isolated by aerobic cultivation on 5 % sheep blood-enriched Columbia agar (BioMérieux, Marcy l’Etoile, France). As the 16S rRNA gene sequence cannot be used as a means of identifying *Pantoea* species, a comparative *rpoB* nucleotide sequences analysis between strain FF5 and other *Pantoea* species was performed. Strain FF5 exhibited a 99.7 % sequence identity with *P. septica*, its phylogenetically closest validly published *Pantoea* species (Fig. 1) [8]. This strain is motile and its cells grown on agar are Gram-negative rods (and have a mean diameter of 0.79-1.06 μm and a mean length of 1.25-2.04 μm).

**Table 1:** Classification and general features of *Pantoea septica* strain FF5 according to the MIGS recommendations [12]

MIGS ID	Property	Term	Evidence code^a^
	Classification	Domain: *Bacteria*	TAS [24]
		Phylum: *Proteobacteria*	TAS [25, 26]
		Class: *Gammaproteobacteria*	TAS [26, 27]
		Order: *Enterobacteriales*	TAS [28]
		Family: *Enterobacteriaceae*	TAS [4, 28, 29]
		Genus: *Pantoea*	TAS [1]
		Species*: Pantoea septica*	IDA
		Strain: FF5	IDA
	Gram stain	Negative	IDA
	Cell shape	Rods	IDA
	Motility	Motile	IDA
	Sporulation	Non-spore forming	IDA
	Temperature range	Mesophile	IDA
	Optimum temperature	37–45 °C	IDA
	pH range; Optimum	6.2–7.5; 6.8	
	Carbon source	Unknown	
MIGS-6	Habitat	Human skin	IDA
MIGS-6.3	Salinity	Growth in BHI medium + 5 % NaCl	IDA
MIGS-22	Oxygen requirement	Aerobic	IDA
MIGS-15	Biotic relationship	Free-living	IDA
MIGS-14	Pathogenicity	Unknown	
MIGS-4	Geographic location	Senegal	IDA
MIGS-5	Sample collection time	December 2012	IDA
MIGS-4.1	Latitude	13.7167	IDA
MIGS-4.1	Longitude	−16.4167	IDA
MIGS-4.4	Altitude	45 m above sea level	IDA

^a^Evidence codes - IDA: Inferred from Direct Assay; TAS: Traceable Author Statement (i.e., a direct report exists in the literature); NAS: Non-traceable Author Statement (i.e., not directly observed for the living, isolated sample, but based on a generally accepted property for the species, or anecdotal evidence). These evidence codes are from the Gene Ontology project [30]

Strain FF5 was catalase-positive but oxidase-negative. Using the API 20E system (BioMérieux), positive reactions were detected for β-galactosidase, citrate, tryptophan deaminase, mannitol, inositol, rhamnose, saccharose, melibiose, arabinose and sorbitol. Negative reactions were noted for arginine dehydrolase, lysine decarboxylase, hydrogen sulfide (H_2_S), urease, indole and amygdalin. Using API 50 CH (BioMérieux), positive reactions were observed for glycerol, D-ribose, D-xylose, D-galactose, D-glucose, D-fructose, D-mannose, D-maltose, D-trehalose, D-lyxose and D-fucose. Negative reactions were observed for erytritol, L-xylose, D-adonitol, methyl β-D-xylopyranoside, L-sorbose, dulcitol, methyl α-D-mannopyranoside, methyl α-D-glucopyranoside, arbutine, salicin, D-cellobiose, inulin, D-melezitose, starch, potassium gluconate, glycogen and 5-keto-D-gluconate. Using API ZYM, positive reactions were observed for alkaline phosphatase, esterase (C4), esterase lipase (C8), leucine arylamidase, acid phosphatase, naphthol-AS-BI-phosphohydrolase and . Negative reactions were observed for valine arylamidase, trypsin, α-chrymotrypsin, α-galactosidase, α-glucosidase, β-glucosidase, N-acetyl-β-glucosaminidase, α-mannosidase and α-fucosidase. Strain FF5 is susceptible to ceftriaxone, imipenem, gentamicin and ciprofloxacin but resistant to penicillin, amoxicillin, ticarcillin, amoxicillin-clavulanic acid, trimethoprim-sulfamethoxazole, colistin and vancomycin. Thus, the phenotypic characteristics of this strain support the claim that it belongs to *Pantoea septica*.

**Fig. 2:**
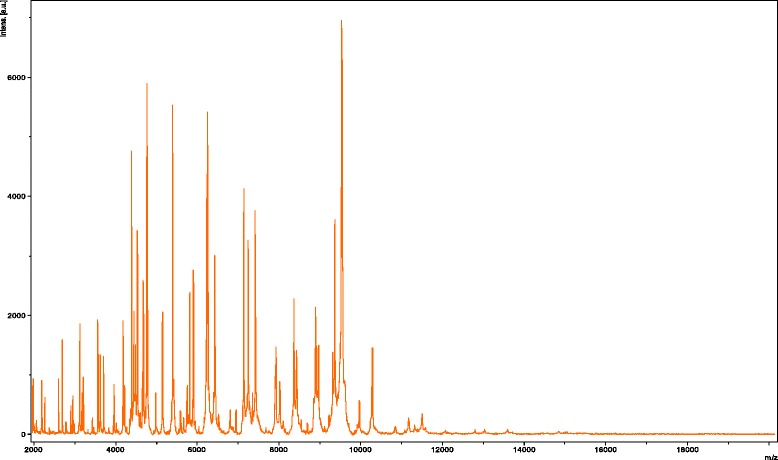
Reference mass spectrum from *Pantoea septica* strain FF5. Spectra from 12 individual colonies were analyzed and a reference spectrum was generated

Matrix-assisted laser-desorption/ionization time-of-flight mass spectrometry protein analysis was performed using a Microflex spectrometer (Bruker Daltonics, Leipzig, Germany), as previously reported [9]. The scores previously established by Bruker Daltonics, used to validate or invalidate identification compared to the instrument database, were applied. Briefly, a score ≥ 2 for a species with a validly published name provided allows the identification at the species level; a score ≥ 1.7 and < 2 allows the identification at the genus level; and a score < 1.7 does not allow any identification. Twelve distinct deposits of strain FF5 were made from 12 isolated colonies. Each smear was overlaid with 2 μL of matrix solution (saturated solution of alpha-cyano-4-hydroxycinnamic acid) and dried for 5 min, as previously reported [9, 10]. The spectra from the 12 different colonies were imported into the MALDI BioTyper software (version 2.0, Bruker) and analyzed by standard pattern matching (with default parameter settings) against the spectra of 6252 bacterial spectra. Spectra were compared with the Bruker database that contained spectra from the ten validly named *Pantoea* species. The spectra obtained were similar to those of *P. septica*. A score of 2.3 was obtained for strain FF5 supporting the identification of *P. septica*. Its reference mass spectrum was added to our database (Fig. 2).

## Genome sequencing information

### Genome project history

*Pantoea septica* strain FF5 was selected for sequencing because no genome of *P. septica* has previously been described. Besides, this strain is part of a study aiming to characterize the skin flora of healthy Senegalese people. It is the 17^th^ genome of *Pantoea* species to be sequenced and the first genome within *P. septica*. The GenBank accession number is CCAQ000000000 and it consists of 4 scaffolds and 37 contigs. Table 2 shows the project information and its association with MIGS version 2.0 compliance [11]. Associated MIGS records are detailed in Additional file 1: Table S1.

**Table 2:** Project information

MIGS ID	Property	Term
MIGS-31	Finishing quality	High-quality draft
MIGS-28	Libraries used	Paired-end and mate-pair 9-kb library
MIGS-29	Sequencing platforms	MiSeq
MIGS-31.2	Fold coverage	26×
MIGS-30	Assemblers	Newbler version 2.5.3
MIGS-32	Gene calling method	Prodigal
	Locus Tag	Not indicated
	Genbank ID	CCAQ000000000
	Genbank Date of Release	March 18, 2014
	GOLD ID	Gp0100998
	BioProject ID	PRJEB4277
MIGS-13	Source material identifier	DSM 27843
	Project relevance	Study of human skin flora

### Growth conditions and genomic DNA preparation

*Pantoea septica* strain FF5 (= CSUR P3024 = DSM 27843) was grown aerobically on 5 % sheep blood-enriched Columbia agar (bioMérieux) at 37 °C. Bacteria grown on four Petri dishes were resuspended in 5 × 100 μL of TE buffer; 150 μL of this suspension was diluted in 350 μL 10X TE buffer, 25 μL proteinase K and 50 μL sodium dodecyl sulfate for lysis treatment. This preparation was incubated overnight at 56 °C. DNA was purified using 3 successive phenol-chloroform extractions and ethanol precipitation at −20 °C of at least two hours each. Following centrifugation, the DNA was suspended in 65 μL EB buffer. Genomic DNA concentration was measured at 46.06 ng/μL using the Qubit assay with the high-sensitivity kit (Life technologies, Carlsbad, CA, USA).

### Genome sequencing and assembly

The genomic DNA of *Pantoea septica* was sequenced using MiSeq Technology (Illumina Inc, San Diego, CA, USA) with the 2 applications: paired-end and mate-pair. The paired-end and mate-pair strategies were barcoded in order to be mixed respectively with 10 other genomic projects prepared with the Nextera XT DNA sample prep kit (Illumina) and 11 other projects with the Nextera Mate-Pair sample prep kit (Illumina).

Genomic DNA was diluted to 1 ng/μL to prepare the paired-end library. The “tagmentation” step fragmented and tagged the DNA with an optimal size distribution of 2.25 kb. Limited cycle PCR amplification (12 cycles) completed the tag adapters and introduced dual-index barcodes. After purification on AMPure XP beads (Beckman Coulter Inc, Fullerton, CA, USA), the libraries were normalized on specific beads according to the Nextera XT protocol (Illumina). Normalized libraries were pooled into a single library for sequencing on the MiSeq. The pooled single-strand library was loaded onto the reagent cartridge, then onto the instrument along with the flow cell. Automated cluster generation and paired-end sequencing with dual index reads were performed in single 39-h run in 2x250-bp. Total information of 5.91 GB was obtained from a 654 K/mm2 cluster density with a cluster passing quality control filters of 93.7 % (12,204,000 clusters). Within this run, the index representation for *P. septica* was determined to be 2.25 %. So *P. septica* has 257,400 reads filtered according to the read qualities.

The mate pair library was prepared with 1 μg of genomic DNA using the Nextera mate-pair Illumina guide. The genomic DNA sample was simultaneously fragmented and tagged with a mate-pair junction adapter. The fragmentation profile was validated on an Agilent 2100 BioAnalyzer (Agilent Technologies Inc, Santa Clara, CA, USA) with a DNA 7500 labchip. The DNA fragments ranged in size from 1.5 kb up to 14 kb with an optimal size of 9 kb. No size selection was performed and 600 ng of tagmented fragments were circularized. The circularized DNA was mechanically sheared into small fragments on the Covaris device S2 in microtubes (Covaris, Woburn, MA, USA). The library profile was visualized on a High-Sensitivity Bioanalyzer LabChip (Agilent Technologies Inc, Santa Clara, CA, USA). The libraries were normalized at 2 nM and pooled. After a denaturation step and dilution to 10 pM, the pool of libraries was loaded onto the reagent cartridge, then onto the instrument along with the flow cell. Automated cluster generation and sequencing were performed in a single 39-h run in a 2x250-bp.

An overall quantity of 3.2 GB was obtained from a 690 K/mm2 cluster density with a cluster passing quality control filters of 95.4 % (13,264,000 clusters). The index representation for *P. septica* was determined to be 7.26 % within this run. *P. septica* has a total of 918,753 reads filtered according to the read qualities.

### Genome annotation

Open Reading Frames prediction was performed using Prodigal [12] with default parameters. We removed the predicted ORFs if they spanned a sequencing gap region. Functional assessment of protein sequences was performed by comparing them with sequences in the GenBank [13] and Clusters of Orthologous Groups (COG) databases using BLASTP. tRNAs, rRNAs, signal peptides and transmembrane helices were identified using tRNAscan-SE 1.21 [14], RNAmmer [15], SignalP [16] and TMHMM [17] respectively. Artemis [18] was used for data management whereas DNA Plotter [19] was used for visualization of genomic features. In-house perl and bash scripts were used to automate these routine tasks. ORFans were sequences with no homology in a given database i.e. in a non-redundant (nr) or identified if their BLASTP E-value was lower than 1e-03 for alignment lengths greater than 80 amino acids. If alignment lengths were smaller than 80 amino acids, we used an E-value of 1e-05. PHAST was used to identify, annotate and graphically display prophage sequences within bacterial genomes or plasmids [20].

To estimate the nucleotide sequence similarity at the genome level between *P. septica* and another 7 members of the genus of *Pantoea* and 4 members of the genus *Enterobacter*, we determined the AGIOS parameter as follows: orthologous proteins were detected using the Proteinortho software (with the parameters following: E-value 1e-5, 30 % identity, 50 % coverage and algebraic connectivity of 50 %) [21] and genomes compared two by two. After fetching the corresponding nucleotide sequences of orthologous proteins for each pair of genomes, we determined the mean percentage of nucleotide sequence identity using the Needleman-Wunsch global alignment algorithm. The script created to calculate AGIOS values was named MAGi (Marseille Average genomic identity) and is written in perl and bioperl modules. GGDC analysis was also performed using the GGDC web server as previously reported [22].

## Genome properties

**Fig. 3:**
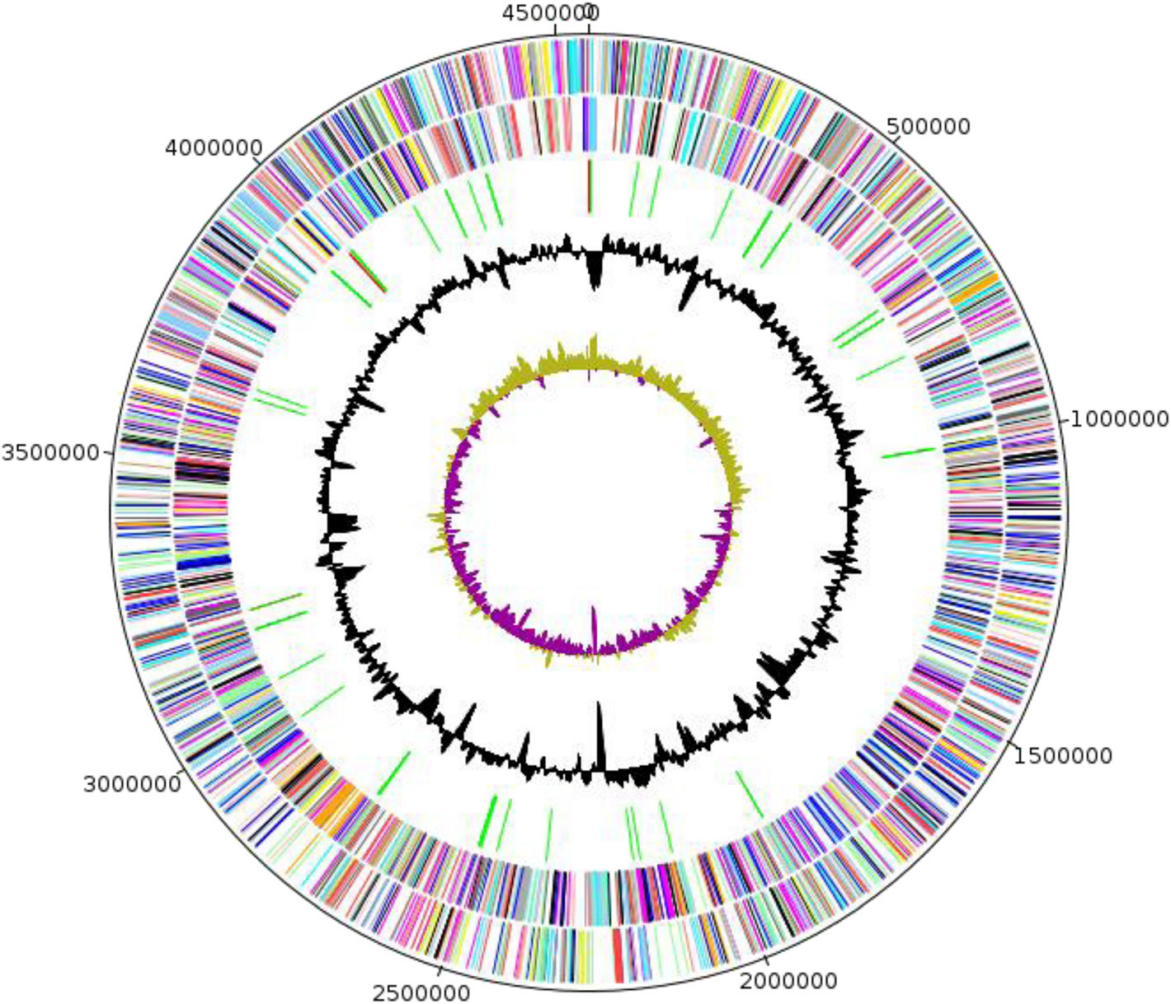
Graphical circular map of the chromosome of *P. septica* strain FF5. From the outside in the two outer circles show open reading frames oriented in the forward (colored by COG categories) and reverse (colored by COG categories) directions, respectively. The third circle marks the rRNA gene operon (red) and tRNA genes (green). The fourth circle shows the G + C% content plot. The innermost circle shows GC skew, with purple and olive indicating negative and positive values, respectively

The genome of *P. septica* strain FF5 is 4,548,444 bp long (1 chromosome, no plasmid) with a 59.1 % G + C content (Fig. 3). Of the 4193 predicted genes, 4125 were protein-coding genes and 68 were RNAs. A total of 3040 genes (72.50 %) were assigned a putative function. A total of 522 genes were annotated as hypothetical proteins. The properties and statistics of the genome are presented in Table 3. The distribution of genes into COG functional categories is presented in Table 4. A total of 214 were identified as ORFans (5.18 %).

**Table 3:** Nucleotide content and gene count levels of the genome

Attribute	Value	% of total^a^
Genome size (bp)	4,548,444	
DNA coding (bp)	3,981,573	87.54
DNA G + C (bp)	2,687,917	59.1
DNA scaffolds	4	-
Total genes	4,193	100.00
Protein-coding genes	4,125	98.37
RNA genes	68	1.50
Pseudo genes	22	0.53
Genes in internal clusters	N/D^b^	-
Genes with function prediction	3,040	72.50
Genes assigned to COGs	3,562	84.97
Genes with Pfam domains	134	3.24
Genes with peptide signals	214	5.18
Genes with transmembrane helices	1,026	24.87
ORFan genes	532	12.89
CRISPR repeats	3	

^a^The total is based on either the size of genome in base pairs or the total number of protein-coding genes in the annotated genome

**Table 4:** Number of genes associated with general COG functional categories

Code	Value	% age	Description
J	173	4.19	Translation, ribosomal structure and biogenesis
A	1	0.02	RNA processing and modification
K	274	6.64	Transcription
L	118	2.86	Replication, recombination and repair
B	0	0.00	Chromatin structure and dynamics
D	33	0.80	Cell cycle control, Cell division, chromosome partitioning
V	41	0.99	Defense mechanisms
T	99	2.40	Signal transduction mechanisms
M	191	4.63	Cell wall/membrane biogenesis
N	41	0.99	Cell motility
Z	0	0.00	Cytoskeleton
U	34	0.82	Intracellular trafficking and secretion
O	113	2.73	Posttranslational modification, protein turnover, chaperones
C	186	4.50	Energy production and conversion
G	233	5.65	Carbohydrate transport and metabolism
E	309	7.49	Amino acid transport and metabolism
F	75	1.82	Nucleotide transport and metabolism
H	113	2.74	Coenzyme transport and metabolism
I	69	1.67	Lipid transport and metabolism
P	207	5.01	Inorganic ion transport and metabolism
Q	36	0.87	Secondary metabolite biosynthesis, transport and catabolism
R	363	8.80	General function prediction only
S	331	8.02	Function unknown
-	522	12.65	Not in COGs

The total is based on the total number of protein-coding genes in the annotated genome

## Insights from genome sequence

Here, we compared 11 genome sequences including *Pantoea ananatis* strain LMG 20103, *P. vagans* strain C9-1, *P. ananatis* strain LMG 5342, *P. ananatis* strain AJ13355, *P. ananatis* strain PA13, *P. agglomerans* strain 299R, *P. stewartii* subsp. *stewartii* strain DC283, *Enterobacter cloacae* subsp. *dissolvens* strain SDM, *E. aerogenes* strain EA1509E, *E. asburiae* strain LF7a and *E. cloacae* strain EcWSU1 (Table 5).

**Table 5:** Comparison of *Pantoea septica* strain FF5 with other genomes of several *Pantoea* species and some *Enterobacter* species

Microorganisms used for genome comparison	Accession number	Genome size (bp)	GC%	Number of proteins
*P. septica* strain FF5	CCAQ000000000	4,548,444	59.10	4,125
*P. ananatis* strain LMG 20103	NC_013956	4,703,373	53.69	4,241
*P. vagans* strain C9-1	NC_014562	4,024,986	55.55	3,664
*P. ananatis* strain LMG 5342	NC_016816	4,605,545	53.45	4,324
*P. ananatis* strain AJ13355	NC_017531	4,555,536	53.76	3,760
*P. ananatis* strain PA13	NC_017554	4,586,378	53.66	4,130
*P. agglomerans* strain 299R	ANKX00000000	4,581,483	54.30	4,157
*P. stewartii* subsp. *stewartii* strain DC283	AHIE000000000	5,233,214	53.80	4,903
*E. cloacae* subsp. *dissolvens* strain SDM	NC_018079	4,968,248	55.06	4,542
*E. aerogenes* strain EA1509E	NC_020181	5,419,609	54.98	5,260
*E. asburiae* strain LF7a	NC_015968	4,812,833	53.85	4,409
*E. cloacae* strain EcWSU1	NC_016514	4,734,438	54.61	4,534

Table 5 shows a comparison of genome size, G + C content, coding-density and number of proteins for these genomes.

The G + C content (59.1 %) of *P. septica* strain FF5 differed by more than 1 % from all other compared species within the genus *Pantoea* [*P. vagans* strain C9-1 (55.55), *P. ananatis* strains LMG 5342, AJ13355 and PA13 (53.45, 53.76, and 53.66, respectively), *P. agglomerans* strain 299R (54.3), *P. stewartii* subsp. *stewartii* strain DC283 (53.8)].

According to the previous demonstration that the G + C content deviation is at most 1 % within species, these values confirm the classification of strain FF5 in a distinct species [23].

Orthologous gene comparison of *P. septica* strain FF5 with other closely related species are summarized in Table 6. Intraspecies values ranged from 99.06 to 99.33 % for *P. ananatis* (Table 7). Interspecies AGIOS values ranged from 77.46 to 84.94 % within the *Pantoea* genus, and from 71.27 to 72.57 % between *Pantoea* and *Enterobacter* species (Table 7). When compared to other species, *P. septica* exhibited AGIOS values ranging from 77.7 to 80.5 with *Pantoea* species and from 72.38 to 73.26 with *Enterobacter* species (Table 7).

**Table 6:** Orthologous gene comparison of *Pantoea septica* strain FF5 with other closely related species

	*P. septica*	*P. agglomerans*	*P. stewartii*	*P. ananatis* LMG20103	*P. vagans* C9	*P. ananatis* LMG5342	*P. ananatis* AJ13355	*P. ananatis* PA13	*E. cloacae* SDM	*E. aerogenes* EA1509E	*E. asburiae* ELF7a	*E. cloacae* EcWSU1
*P. septica*	**4,125**											
*P. agglomerans*	2,948	**4,157**										
*P. stewartii*	2,677	2,581	**4,903**									
*P. ananatis* LMG20103	2,993	2,953	3,024	**4,241**								
*P. vagans*-C9	2,928	2,889	2,576	2,889	**3,664**							
*P. ananatis*-LMG5342	2,868	2,792	2,917	3,527	2,852	**4,324**						
*P. ananatis* AJ13355	2,778	2,698	2,775	3,372	2,752	3,413	**3,760**					
*P. ananatis* PA13	2,876	2,801	2,960	3,560	2,883	3,648	3,402	**4,130**				
*E. cloacae* SDM	2,736	2,536	2,400	2,688	2,535	2,586	2,549	2,585	**4,542**			
*E. aerogenes* EA1509E	2,688	2,495	2,400	2,672	2,528	2,617	2,570	2,612	3,282	**5,260**		
*E. asburiae* ELF7a	2,634	2,471	2,393	2,634	2,502	2,577	2,542	2,588	3,650	3,249	**4,409**	
*E. cloacae* EcWSU1	2,674	2,526	2,387	2,664	2,529	3,456	2,558	2,520	2,572	3,457	3,105	**4,534**

Bold numbers indicate the number of genes from each genome

**Table 7:** dDDH values (upper right) and AGIOS values (lower left) obtained by comparison of all studied genomes

	*P. septica*	*P. ananatis* LMG 20103	*P. vagans* C9-1	*P. ananatis* LMG 5342	*P. ananatis* AJ13355	*P. ananatis* A13	*P. agglomerans* 299R	*P. stewartii* DC283	*E. cloacae* SDM	*E. aerogenes* EA1509E	*E. asburiae* LF7a	*E. cloacae*EcWSU1
*P. septica*		0.2038	0.1913	0.2041	0.2036	0.2033	0.1966	0.203	0.2152	0.2091	0.2182	0.2174
*P. ananatis* LMG 20103	77.7		0.1916	0.0084	0.0077	0.0089	0.1955	0.1534	0.2127	0.2026	0.2158	0.2151
*P. vagans* C9-1	80.5	79.69		0.1907	0.1908	0.1907	0.0935	0.191	0.214	0.2126	0.2121	0.2125
*P. ananatis* LMG 5342	78.11	99.14	79.85		0.0094	0.0099	0.1956	0.1519	0.2136	0.2027	0.2177	0.2133
*P. ananatis* AJ13355	78.17	99.33	79.96	99.33		0.009	0.1959	0.1523	0.2144	0.2032	0.2176	0.2131
*P. ananatis*PA13	78.06	99.07	79.81	99.07	99.11		0.196	0.1519	0.2145	0.2032	0.216	0.2139
*P. agglomerans* 299R	79.12	78.75	91.2	79.14	79.22	78.06		0.1973	0.2197	0.2207	0.2208	0.222
*P. stewartii* DC283	78.01	84.54	79.79	84.73	84.94	84.6	78.99		0.2136	0.2025	0.2183	0.2134
*E. cloacae* SDM	72.79	71.6	72.57	71.64	71.79	71.68	71.92	71.22		0.1917	0.1379	0.1194
*E. aerogenes* EA1509E	73.26	71.48	72.37	71.44	71.58	71.41	71.76	71.53	78.09		0.1955	0.195
*E. asburiae* LF7a	72.38	71.38	72.22	71.34	71.44	71.27	71.77	71.52	85.85	77.73		0.1394
*E. cloacae* EcWSU1	72.68	71.52	72.38	85.73	71.59	71.74	71.76	71.67	71.53	87.91	78.38	

## Conclusions

We describe the genome of *Pantoea septica* strain FF5. This is the first reported genome of *P. septica*. We also report phenotypic and phylogenetic characteristics of strain FF5. *P. septica* strain FF5 was isolated from the skin flora of a 35-year-old healthy Senegalese woman. The *P. septica* strain FF5 genome sequences are deposited in GenBank under accession number CCAQ000000000.

## Additional file

Additional file 1: Table S1. Associated MIGS record. (DOC 70 kb)

## Abbreviations

DSM: Deutsche Sammlung von Mikroorganismen
CSUR: Collection de Souches de l’Unité des Rickettsies
MALDI: Matrix Assisted Laser Desorption Ionization
AGIOS: Average Genomic Identity of Orthologous Gene Sequences
GGDC: Genome-to-Genome Distance Calculator
dDDH: Digital DNA-DNA hybridization
MIGS: Minimum Information about a Genome Sequence
