# Supplementary material for: Genome sequence and description of Pantoea septica strain FF5
Source: Stand Genomic Sci. 2015 Nov 14;10:103. doi: 10.1186/s40793-015-0083-0 (PMC4644298; doi:10.1186/s40793-015-0083-0)
Supplement: Additional file 1: Table S1. — Associated MIGS record. (DOC 70 kb) [file 40793_2015_83_MOESM1_ESM.doc]

**Additional file 1: Table S1. Associated MIGS record.**

| **MIGS-ID** | field name | Description |
| --- | --- | --- |
| **MIGS-1** | Submit to INSDC/Trace archives | Not reported |
| **1.1** | PID | Not reported |
| **1.2** | Trace Archive | Not reported |
| **MIGS-2** | MIGS CHECK LIST TYPE | Not reported |
| **MIGS-3** | Project Name |  |
| **MIGS-4** | Geographic Location | Dielmo, Senegal |
| **4.1** | Latitude | 13.7167 |
| **4.2** | Longitude | -16.4167 |
| **4.3** | Depth | Surface |
| **4.4** | Altitude | 0 m |
| **MIGS-5** | Time of Sample collection | December 2012 |
| **MIGS-6** | Habitat (EnvO) | Human skin |
| **6.1** | Temperature | 37°C |
| **6.2** | pH | Not reported |
| **6.3** | Salinity | Not reported |
| **6.4** | Chlorophyll | Not reported |
| **6.5** | Conductivity | Not reported |
| **6.6** | Light intensity | Not reported |
| **6.7** | dissolved organic carbon (DOC) | Not reported |
| **6.8** | Current | Not reported |
| **6.9** | Atmospheric data | Not reported |
| **6.10** | Density | Not reported |
| **6.11** | Alkalinity | Not reported |
| **6.12** | dissolved oxygen | Not reported |
| **6.13** | particulate organic carbon (POC) | Not reported |
| **6.14** | Phosphate | Not reported |
| **6.15** | Nitrate | Not reported |
| **6.16** | Sulfates | Not reported |
| **6.17** | Sulfides | Not reported |
| **6.18** | primary production | Not reported |
| **MIGS-7** | Subspecific genetic lineage | Not reported |
| **MIGS-9** | Number of replicons | 1 |
| **MIGS-10** | Extrachromosomal elements | 0 |
| **MIGS-11** | Estimated Size | 4,548,444 bp |
| **MIGS-12** | Reference for biomaterial or Genome report | Not reported |
| **MIGS-13** | Source material identifiers | Not reported |
| **MIGS-14** | Known Pathogenicity | Not reported |
| **MIGS-15** | Biotic Relationship | Not reported |
| **MIGS-16** | Specific Host | Not reported |
| **MIGS-17** | Host specificity or range (taxid) | Not reported |
| **MIGS-18** | Health status of Host | healthy patient |
| **MIGS-19** | Trophic Level | Not reported |
| **MIGS-22** | Relationship to Oxygen | Aerobic |
| **MIGS-23** | Isolation and Growth conditions | 5% sheep blood agar at 37°C |
| **MIGS-27** | Nucleic acid preparation | EZ1 DNA Tissue kit (Qiagen) |
| **MIGS-28** | Library construction | 454 paired end 3-kb library (Roche) |
| **28.1** | Library size |  |
| **28.2** | Number of reads |  |
| **28.3** | Vector |  |
| **MIGS-29** | Sequencing method | Miseq |
| **MIGS-30** | Assembly | Newbler software (Roche) |
| **30.1** | Assembly method | Not reported |
| **30.2** | estimated error rate | Not reported |
| **30.3** | method of calculation | Not reported |
| **MIGS-31** | Finishing strategy | Not reported |
| **31.1** | Status | Draft genome, unfinished |
| **31.2** | Coverage | 26x |
| **31.3** | Contigs | 37 |
| **MIGS-32** | Relevant SOPs | Not reported |
| **MIGS-33** | Relevant e-resources | Not reported |
